# Umbilical cord ulceration associated with placental abruption: a case report

**DOI:** 10.1186/s12884-026-09124-y

**Published:** 2026-05-08

**Authors:** Yanqing Peng, Guannan He, Chuanju Zhang, Litao Sun, Jing Zhao

**Affiliations:** 1 Cancer Center, Department of Ultrasound Medicine, Zhejiang Provincial People’s Hospital (Affiliated People’s Hospital), Hangzhou Medical College, Hangzhou, Zhejiang China; 2 Department of Ultrasound Medicine, Sichuan Provincial Maternity and Child Health Care Hospital, Chengdu, Sichuan China; 3 Sir Run Run Shaw Hospital, School of Medicine, Zhejiang University, Hangzhou, Zhejiang China

**Keywords:** Umbilical cord ulceration, Placental abruption, Prenatal ultrasound, Wharton’s jelly, Case report

## Abstract

**Background:**

Umbilical cord ulceration (UCU) is a rare but potentially fatal obstetric condition characterized by ulcerative disruption of the umbilical cord, often leading to fetal hemorrhage and intrauterine fetal demise. Placental abruption, defined as the premature separation of the placenta from the uterine wall, is another major cause of perinatal mortality. When UCU is complicated by occult placental abruption, the risk of adverse perinatal outcomes increases substantially. These associations highlight the importance of vigilant prenatal surveillance and comprehensive imaging to facilitate early detection and timely intervention.

**Case presentation:**

We report a case involving a 33-year-old woman at 37 weeks and 5 days of gestation who presented with abdominal tightness and subjectively reduced fetal movements. Prenatal ultrasound and Color Doppler imaging (CDI) revealed umbilical cord abnormalities, including loss of Wharton’s jelly and absent blood flow signals. An emergency cesarean section was promptly performed, resulting in favorable maternal and neonatal outcome. Postoperative pathological examination confirmed the diagnosis of UCU with hemorrhage, complicated by placental abruption.

**Conclusions:**

To our knowledge, this is the first reported case of UCU complicated by placental abruption. This case underscores the importance of considering UCU in the differential diagnosis of unexplained fetal distress in late pregnancy. Early recognition and timely intervention guided by prenatal imaging are essential for improving perinatal outcomes.

## Background

Umbilical cord ulceration (UCU) is a rare prenatal condition characterized by localized degeneration and loss of Wharton’s jelly with exposure of the umbilical vessels. This defect may lead to vascular rupture and severe fetal hemorrhage, particularly when associated with intestinal atresia or premature rupture of membranes (PPROM), and is considered potentially fatal [1, 2]. Although the overall incidence of UCU remains uncertain, studies have reported a prevalence of 6.5% to 13.6% among fetuses with intestinal atresia [3]. A literature review of 38 cases demonstrated a fetal mortality rate of 32%, a neonatal mortality rate of 32%, and an overall survival rate of only 37% [2]. Placental abruption, defined as the premature separation of the placenta from the uterine wall, is typically characterized by vaginal hemorrhage, uterine tenderness, and abnormal fetal heart rate patterns. It occurs in approximately 0.6% to 1.2% of all pregnancies and accounts for 10% to 20% of perinatal deaths, remaining one of the leading causes of fetal demise [4, 5]. Early recognition of UCU is often challenging due to its nonspecific prenatal presentation. Perinatal mortality increases significantly when UCU is complicated by occult placental abruption, underscoring the importance of vigilant antenatal surveillance and comprehensive prenatal imaging to mitigate the risk of adverse perinatal outcomes.

Here, we report a case of UCU with hemorrhage complicated by placental abruption in a young woman during late pregnancy. To our knowledge, this is the first reported case of UCU presenting with hemorrhage in combination with placental abruption during late pregnancy. Most previously reported cases of UCU have occurred in preterm pregnancies and were associated with preterm PPROM and congenital intestinal atresia [1–3, 6]. In contrast, the present case occurred in late pregnancy without gastrointestinal malformations or PPROM, underscoring the uncommon and distinctive clinical presentation of UCU. This case highlights the critical role of integrating comprehensive imaging findings with clinical presentation for accurate diagnosis. The present report aims to raise clinical awareness of this rare condition that carries a potential risk of being life-threatening, and to emphasize the importance of early recognition and timely intervention to improve perinatal outcomes.

## Case presentation

A 33-year-old woman at 37 weeks and 5 days of gestation presented with complaints of abdominal tightness lasting over 10 h and a marked reduction in perceived fetal movements. Based on the clinical presentation, placental abruption was initially suspected, and the patient was promptly referred to the emergency department of our hospital for further evaluation and management. Physical examination revealed mild abdominal tension without evidence of vaginal hemorrhage or amniotic fluid leakage. Obstetric assessment indicated that the fetal presenting part was not engaged in the maternal pelvis. The fetal heart rate was 150 beats per minute, and the membranes remained intact. Cardiotocography showed mild baseline variability with late decelerations and irregular uterine contractions. Previous routine prenatal evaluations, including an ultrasound performed two weeks earlier at our hospital, had been unremarkable.

**Fig. 1:**
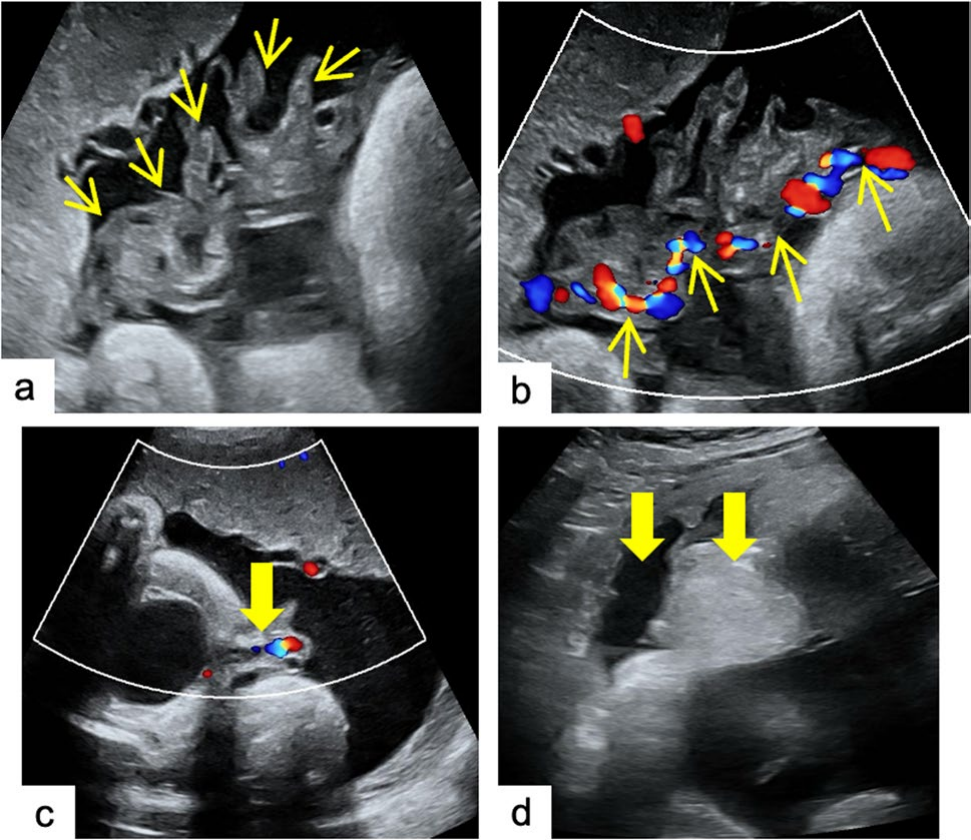
Prenatal ultrasound and Color Doppler imaging (CDI) findings. **a** Disorganization of the umbilical cord structure, with flocculent hypoechoic and mildly echogenic material floating in the surrounding amniotic fluid (arrows). **b** CDI demonstrates attenuated umbilical vessels and surrounding avascular flocculent zones (arrows). **c** The cord insertion site shows a thinned umbilical artery and a wrinkled, discontinuous amniotic membrane with weak internal echoes (arrow). **d** A large anechoic area between the placenta and uterine wall, containing flocculent material (arrows)

Emergency ultrasound revealed disorganization of the umbilical cord architecture and loss of its normal morphology. Visualization was limited due to inadequate delineation of Wharton’s jelly. Flocculent hypoechoic and mildly echogenic material was observed floating in the amniotic fluid surrounding the umbilical cord (Fig. 1a). Color Doppler imaging (CDI) demonstrated attenuated umbilical arteries and vein, accompanied by peripheral hypoechoic flocculent regions without detectable blood flow signals (Fig. 1b). Further ultrasonography revealed that the umbilical artery at the site of insertion into the fetal abdominal wall was narrowed in caliber. The overlying amniotic membrane appeared wrinkled, discontinuous, and flocculent, with weak internal echogenicity (Fig. 1c). A large, patchy anechoic area measuring 8.8 × 1.3 cm was identified between the fetal membranes and the anterior uterine wall, containing flocculent, mildly echogenic material—a typical ultrasonographic feature of a retroplacental hematoma, and findings suggestive of placental abruption with associated intrauterine hemorrhage (Fig. 1d).

**Fig. 2:**
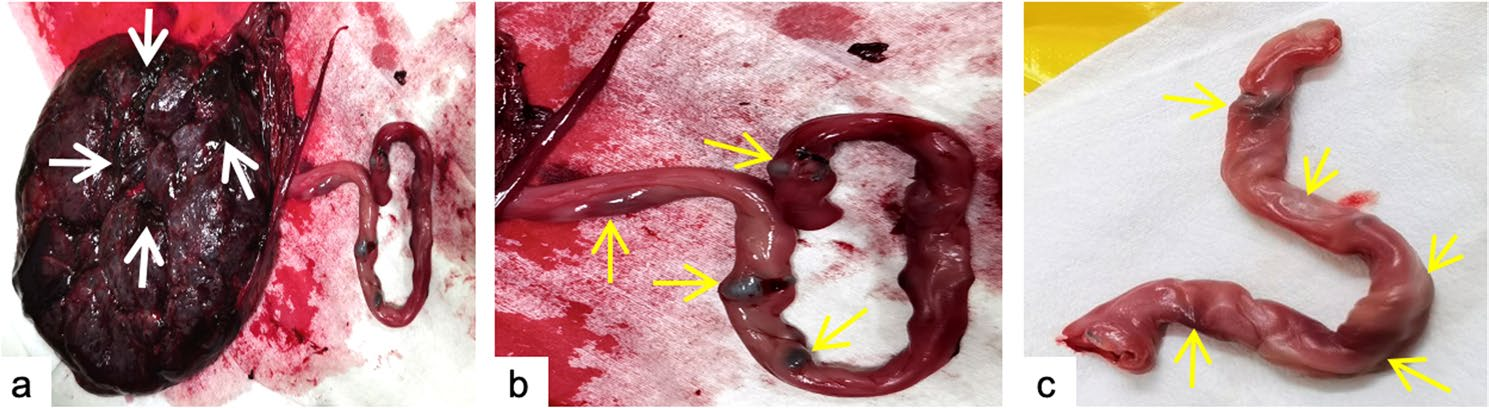
Gross findings of the umbilical cord and placenta.** a** The umbilical cord appears slender, with multiple surface ulcers and exposed vessels. **b**,** c** Multiple rupture sites showing disruption of the vessel walls, thrombus formation, and partial absence of Wharton’s jelly and the amniotic membrane (arrows)

**Fig. 3:**
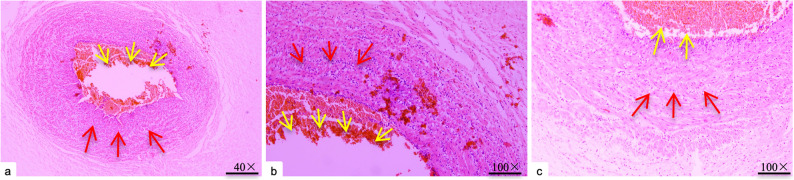
Histopathology findings of the umbilical cord. The umbilical vein shows intraluminal red blood cell infiltration (yellow arrows) and neutrophilic infiltration within the venous wall (red arrows)

The critical findings were promptly communicated to the obstetric emergency team, and an emergency cesarean section was performed. Intraoperatively, about 500 mL of thick, dark red, bloody amniotic fluid was observed. The umbilical cord appeared slender and demonstrated signs of degeneration near its fetal end, with the absence of Wharton’s jelly, multiple ruptures, and exposed umbilical vessels. On the maternal surface of the placenta, a ring-shaped area of old hemorrhagic indentation measuring approximately 15 × 3 cm was noted. An estimated 800 mL of intrauterine blood and a small amount of blood clots were also present within the uterine cavity. Postoperative gross examination of the placenta revealed a hematoma on the maternal surface. The umbilical cord was thin, with multiple ulcerated breaches (Fig. 2a). Wharton’s jelly and the overlying amniotic membrane were partially absent. Multiple sites of cord ruptures were evident, with extensive exposure of the umbilical vessels. The vessel walls were disrupted, and fresh thrombi were identified at the rupture sites (Fig. 2b and c). Histopathological examination revealed placental subchorionic thrombosis, intraplacental hematoma, focal distal chorionic adhesions, and focal distal chorionic interstitial vascularization. Maternal inflammation was consistent with acute chorioamnionitis (stage 1 of 3, grade 2 of 2), while fetal inflammation response manifested as umbilical phlebitis (stage 1 of 3, grade 1 of 2). The umbilical cord exhibited a single-artery and single-vein (1A1V) configuration. Wharton’s jelly was absent, the vessel walls were markedly thinned and necrotic, and the overlying amniotic membrane showed signs of degeneration and necrosis (Fig. 3a-c).

Prenatal ultrasound revealed a retroplacental hematoma typical of placental abruption, aiding in the identification of concealed hemorrhage. Based on the intraoperative findings and the total placental area at term, the detachment was estimated to involve 15%–20% of the surface (< 1/3). The presence of fetal distress was consistent with Grade 2 placental abruption. Based on the imaging findings and histopathological confirmation, a final diagnosis of UCU with hemorrhage and concurrent placental abruption was established.

A female neonate was delivered with a birth weight of 3,270 g. At birth, she exhibited no spontaneous cry, irregular respiration, hypotonia, cyanosis, and an oxygen saturation of 65%. Despite initial corrective ventilation, spontaneous respiration function could not be restored. The Apgar score was 4 at 1 min. Immediate resuscitation measures, including tracheal intubation and positive pressure ventilation, were initiated. The Apgar score subsequently improved to 6 at 5 min and 7 at 10 min. The neonate was then transferred to the Department of Neonatology for further care. Both the mother and the infant eventually achieved favorable clinical recovery.

### Discussion and Conclusion

The umbilical cord comprises two arteries and one vein. These vessels are surrounded by Wharton’s jelly, which provides structural support and protection, and the entire structure is enveloped by the amniotic membrane [7]. Functionally, the umbilical cord connects the fetus to the maternal placenta, facilitating gas exchange, nutrient delivery, and elimination of fetal metabolic waste [7]. UCU is an exceptionally rare condition, that is considered potentially fatal due to the risk of cord vessel rupture and massive fetal hemorrhage, first described by Bendon et al. in 1991 [6]. Prenatal clinical manifestations are often nonspecific and may include reduced fetal movements and abnormal fetal monitoring findings [8]. At delivery, affected neonates frequently present in poor condition, with findings such as bloody amniotic fluid and active hemorrhage from exposed umbilical vessels [9]. In the present case, a young woman at 37 weeks and 5 days of gestation presented with over 10 h of abdominal tightness and a subjective reduction in fetal movements. Subsequent imaging and postoperative pathology confirmed a diagnosis of UCU complicated by placental abruption. Ultimately, both the mother and the neonate had a favorable clinical outcome. This case highlights the importance of considering rare but potentially fatal conditions such as UCU, which carries the risk of umbilical vessel rupture and massive fetal hemorrhage, in the differential diagnosis of obstetric presentations. Early recognition and timely communication of such critical findings are essential for initiating immediate clinical intervention and improving neonatal survival.

The underlying mechanism of UCU remains unclear. Possible etiologic factors include congenital upper gastrointestinal atresia, which may cause reflux of fetal gastrointestinal contents containing digestive enzymes into the amniotic fluid. These enzymes can erode Wharton’s jelly and compromise the integrity of the umbilical cord [2]. Some studies also suggest that fetal meconium may induce vasoconstriction of umbilical vessels and damage to the vascular wall, triggering inflammation and contributing to the development of UCU [7, 10]. As the pathogenesis of UCU involves Wharton’s jelly degeneration or deficiency, degenerative changes in the amniotic membrane, thinning of the umbilical vascular wall, and secondary hemorrhage, several studies have proposed a pathological grading system. This classification divides lesions into four grades: Grade 1, loss of the amniotic epithelium; Grade 2, detachment of the amniotic epithelial basement membrane; Grade 3, loss of Wharton’s jelly or extensive Grade 2-like changes; and Grade 4, exposure of the umbilical artery or vein [3]. Based on the gross and histopathological findings in the present case, the lesion can be classified as Grade 4 UCU, characterized by loss of Wharton’s jelly, multiple cord ruptures, and extensive exposure of the umbilical vessels. Although no prenatal gastrointestinal malformations were identified in this patient, close and regular monitoring of the umbilical cord is essential in late pregnancy to facilitate timely detection and intervention.

Clinically, UCU often presents with nonspecific antenatal features, making early recognition challenging [2, 8]. Given its rarity and potentially catastrophic consequences, clinical awareness of this condition remains limited. The major prognostic concern is massive fetal hemorrhage, which may lead to intrauterine fetal death or severe neonatal anemia if not detected promptly [9, 11]. Reported perinatal mortality rates remain high, emphasizing the need for heightened clinical vigilance and timely intervention when umbilical cord abnormalities are suspected [7]. Certain clinical and imaging findings may provide important diagnostic clues. Decreased fetal movement or abnormal fetal heart rate patterns may represent early warning signs, reflecting fetal distress secondary to umbilical cord vascular compromise [2, 8]. Characteristic ultrasonographic findings include focal cord thinning or discontinuity, loss of Wharton’s jelly, and floating echoes or flocculent material in the amniotic fluid suggestive of hemorrhage or necrotic debris [3, 7, 12]. CDI may reveal reduced or absent blood flow in the affected cord segment or the presence of avascular areas surrounding the cord [3, 9]. When these findings coexist with by abnormal cardiac ovoid patterns or intra-amniotic echogenic foci, UCU should be strongly suspected, prompting urgent multidisciplinary evaluation to prevent catastrophic fetal hemorrhage [2, 9]. Although UCU is rare, increased awareness of umbilical cord pathology and detailed prenatal ultrasonographic assessment of cord morphology can facilitate earlier diagnosis and improve perinatal outcomes [2, 7].

Accurate differentiation from other umbilical cord anomalies is essential. Unlike true umbilical cord knots or torsion, which generally preserve the overall morphology of the cord and the integrity of Wharton’s jelly, UCU is marked by focal or extensive loss of Wharton’s jelly and direct exposure or thinning of the vessel wall [2]. Umbilical cord hematomas usually appear as localized hypoechoic or mixed echogenic masses within the cord, whereas hemorrhages associated with UCU tend to be diffuse and may extend into the amniotic cavity [12]. Pseudocords, a benign variant, maintain normal vascular structure and blood flow, clearly distinguishing them from the vascular injury seen in UCU [13]. In our case, prenatal ultrasonography revealed hallmark imaging features consistent with UCU, including disruption of cord architecture, loss of Wharton’s jelly, attenuation of umbilical vessels, and flocculent echogenic material within the amniotic fluid. These findings closely resembled the previously reported ultrasonographic features of UCU. Collectively, these observations underscore the diagnostic value of combining prenatal imaging with clinical evaluation in suspected cases of UCU.

The patient presented at term with markedly reduced fetal movement and abnormal fetal heart rate patterns, suggestive of acute fetal crisis. A bedside ultrasound examination was immediately performed to identify possible causes such as umbilical cord injury, vascular rupture, or placental abruption and to guide delivery decisions [2, 14]. Current guidelines support prompt ultrasound evaluation when findings may influence clinical management, while emphasizing that imaging should not delay indicated delivery [6, 15, 16]. In this case, the scan was completed within minutes and directly prompted an emergency cesarean section, thereby facilitating rather than delaying intervention. For term pregnancies with a pathological nonstress test (NST) results, targeted ultrasound—including biophysical profile and Doppler assessments—should be performed to clarify the cause of fetal compromise and determine the optimal mode and timing of delivery [15–17]. Although urgent delivery remains the definitive management for severe fetal distress, rapid bedside ultrasound can provide essential diagnostic information without delaying intervention.

In this case, intraoperative findings revealed thick, dark-red amniotic fluid and fresh thrombi on ruptured and ulcerated umbilical vessels, indicating acute fetal hemorrhage superimposed on placental hemorrhage. Although both pathologies may contribute to fetal compromise, the primary indication for emergency cesarean section was the clinical and imaging evidence of placental abruption rather than isolated UCU [4]. Nevertheless, early recognition of umbilical cord morphological abnormalities, together with signs of placental abruption in a term patient presenting with decreased fetal movement and nonreassuring fetal heart rate patterns, facilitated timely intervention through rapid multidisciplinary coordination and expedited delivery [15, 16]. When UCU is suspected without overt placental abruption, severe fetal compromise, or a grade 3 condition, close fetal surveillance, targeted Doppler or biophysical profile assessment, and preparedness for emergency delivery are recommended [14, 15]. However, the presence of UCU alone does not constitute an absolute indication for immediate delivery in the absence of maternal or fetal instability.

Histopathologically, UCU is characterized by focal or extensive degeneration of Wharton’s jelly, thinning or necrosis of the umbilical vessel walls, surface ulceration of the cord, and exposure or rupture of the underlying vasculature, often accompanied by thrombosis and intra-amniotic hemorrhage [2]. Inflammatory changes such as chorioamnionitis or funisitis may also be observed [3]. Currently, there is no established prenatal treatment for UCU. Therefore, early detection through prenatal imaging and timely delivery—generally via emergency cesarean section—remains the cornerstone of management to prevent intrauterine fetal demise [11]. In the present case, pathological examination revealed complete loss of Wharton’s jelly, thinning and necrosis of the umbilical vessel walls, vascular exposure, and thrombosis—findings consistent with advanced-stage UCU.

The exact source of bleeding could not be definitively identified. Intraoperative findings of approximately 500 mL of bloody amniotic fluid and 800 mL of intrauterine blood indicated multiple hemorrhagic sites. Imaging, intraoperative, and pathological evidence suggested that the hemorrhage originated from both UCU and placental abruption. Indicators of UCU included degeneration at the fetal end of the cord with multiple ruptures and loss of Wharton’s jelly, forming a direct communication with the amniotic cavity—an anatomical condition favoring bloody amniotic fluid. The patient’s sudden decrease in fetal movement and fetal heart rate deceleration before admission were consistent with fetal hypoxia secondary to acute hemorrhage. Findings supporting placental abruption included an 8.8 cm × 1.3 cm patchy anechoic area between the membranes and anterior uterine wall on ultrasound, a 15 cm × 3 cm ring-shaped hemorrhagic depression on the placental surface, and pathological confirmation of thrombus formation beneath the chorionic plate—direct evidence of hemorrhage attributable to placental abruption. In summary, intrauterine hemorrhage likely resulted from the combined effects of UCU and placental abruption, with neither identifiable as the predominant source.

Placental abruption involves the premature detachment of the placenta from the uterine wall, resulting in hemorrhage and compromised utero-placental circulation, which can lead to fetal hypoxia or intrauterine demise [5]. Although UCU and placental abruption are distinct pathological entities, they may share overlapping clinical manifestations and potentially interrelated pathophysiological mechanisms. Clinically, UCU may present with meconium-stained amniotic fluid, flocculent echogenic material within the amniotic cavity, and ultrasonographic evidence of cord thinning, discontinuity, or loss of Wharton’s jelly, accompanied by absent or diminished blood flow signals indicative of vascular rupture or intra-amniotic hemorrhage [2, 3, 8, 9]. In contrast, placental abruption typically manifests as uterine tenderness, increased uterine tone, and ultrasonographic evidence of a retroplacental hematoma, often accompanied by nonreassuring fetal heart rate patterns or maternal hemodynamic instability [4, 5]. In rare cases, substantial hemorrhage from UCU may accumulate in the uterine cavity, thereby clinically mimicking or potentially precipitating placental abruption [18]. Conversely, hemodynamic disturbances and inflammatory injury secondary to placental abruption may increase umbilical cord vulnerability and contribute to the development of UCU [2, 19].

Given the occurrence of both UCU and placental abruption within the same pregnancy, a unified pathophysiological mechanism may involve coexisting maternal–placental–umbilical cord vascular perfusion abnormalities and inflammatory injury. Maternal vascular malperfusion (MVM) can lead to hypoxia, reperfusion injury, and vascular wall fragility, thereby compromising the integrity of the umbilical cord wall and Wharton’s jelly while predisposing to placental separation [20, 21]. Infectious or inflammatory processes—such as chorioamnionitis or intra-amniotic infection—may induce localized vascular endothelial injury within the umbilical cord, resulting in amniotic epithelial disruption, weakening of the vascular wall, and subsequent ulceration or vascular rupture, which can in turn precipitate placental abruption [8, 22]. Moreover, an imbalance in the procoagulant–fibrinolytic system—such as maternal or fetal coagulation abnormalities—may promote local microthrombus formation, increase vascular wall stress, and impair normal fibrinolysis [23–25]. Collectively, these alterations can culminate in hemorrhage and tissue injury at the umbilical cord–placental interface. In the present case, gross pathological findings demonstrated concurrent features of severe UCU and placental abruption, suggesting the possibility of a bidirectional pathophysiological relationship. Awareness of this potential association is critical, as the simultaneous occurrence of these two conditions may increase the risk of adverse perinatal outcomes and further complicate prenatal diagnosis.

In conclusion, UCU is a rare obstetric complication that is considered potentially fatal due to the risk of cord vessel rupture and massive fetal hemorrhage, necessitating heightened clinical vigilance. This case illustrates that even in the absence of gastrointestinal malformations, subtle prenatal imaging abnormalities combined with clinical symptoms can raise timely suspicion of UCU. When complicated by concurrent placental abruption, as observed in our case, the diagnostic and clinical management become significantly more challenging. Histopathological examination confirmed the diagnosis and revealed the severity of the vascular injury. Prompt recognition and emergency cesarean delivery are crucial to preventing intrauterine fetal demise. Additionally, this case underscores the need to consider UCU in the differential diagnosis of unexplained fetal distress and highlights the need for individualized management based on the overall condition of both the mother and the fetus to optimize perinatal outcomes.

## Data Availability

All data in this study are accessible to the corresponding author.

## Abbreviations

UCU: Umbilical cord ulceration
CDI: Color Doppler imaging
